# Layer-by-Layer Assembled Polyelectrolytes on Ta_3_N_5_ Pyramid Arrays for Efficient Photoelectrochemical Water Splitting

**DOI:** 10.3390/ijms27188281

**Published:** 2026-09-17

**Authors:** Haoran Zhang, Siqi Sun, Shaoxuan Zhang, Jianjun Liao, Shiwei Lin

**Affiliations:** 1 State Key Laboratory of Tropic Ocean Engineering Materials and Materials Evaluation, School of Materials Science and Engineering, Hainan University, Haikou 570228, China; zirconium0504@hainanu.edu.cn (H.Z.);; 2 Key Laboratory of Agro-Forestry Environmental Processes and Ecological Regulation of Hainan Province, School of Environmental Science and Engineering, Hainan University, Haikou 570228, China

**Keywords:** Ta_3_N_5_ pyramid arrays photoanode, surface modulation, polyelectrolyte

## Abstract

Ta_3_N_5_ is a promising material for overall water splitting due to its suitable band positions and high theoretical photocurrent (12.9 mA cm^−2^). However, pristine Ta_3_N_5_ suffers from significant optical, recombination, and resistive losses, resulting in photocurrents and efficiencies far below theoretical expectations. To address these limitations, this work focuses on enhancing photocurrent and overall performance through structural and morphological engineering and surface functionalization. We investigated the potential of polyelectrolytes, poly(sodium-p-styrenesulfonate) (PSS) with anionic groups and poly(diallyl-dimethylammonium chloride) (PDDA) with cationic groups, as a hole transport layer material in Ta_3_N_5_ pyramid arrays photoelectrodes for enhanced photoelectrochemical water splitting. We synthesized pyramid-shaped Ta_3_N_5_ using Na_2_Ta_2_O_6_ as a template. The polyelectrolyte combined with catalysts was used to modify Ta_3_N_5_ to improve charge separation and injection efficiency. After systematically investigating the effect of the polyelectrolyte modification sequence on the photoelectrode, the champion sample Ta_3_N_5_-PDDA_3_PSS_3_-Co(OH)_x_ delivers a photocurrent density of 7.7 mA cm^−2^ at 1.23 V vs. RHE under simulated solar illumination, which is over 7-fold higher than that of pristine Ta_3_N_5_. This work demonstrates that polymer functionalization of nitride semiconductors enables efficient solar-to-fuel conversion.

## 1. Introduction

Photoelectrochemical (PEC) water splitting is a promising strategy for directly converting solar energy into hydrogen and oxygen. Achieving high energy conversion efficiency depends on three key processes: light absorption, charge separation, and surface injection. Among various semiconductor materials, orthorhombic tantalum nitride (Ta_3_N_5_) has attracted considerable attention. Its standard band gap of 2.1 eV is well suited for visible-light absorption and driving the overall water-splitting reaction. Furthermore, its favorable band-edge positions, relatively low electrical resistivity (~7.3 Ω cm), and long carrier lifetime (~7.3 ps) provide additional advantages, enabling excellent PEC performance as a photoanode [1]. Therefore, it has a theoretical solar-to-hydrogen (STH) efficiency of 15.9% and a maximum photocurrent density close to 13 mA cm^−2^. However, most bare Ta3N5 photoanodes perform far below the theoretical value.

In preparing the Ta_3_N_5_ photoanode, the intermediate materials are tantalum oxide and sodium tantalate, which help build nanoscale morphology. But after nitridation, the photoanode surface is always covered by low-valence Ta or defects. This is considered a serious reason for high onset potential and low photocurrent density. Various surface modification strategies have been shown to enhance performance in this regard. Shao et al. treated tantalum oxide via a two-step sintering method, which significantly reduced defects on the oxide surface [2]. Compared with the pristine sample, the surface electron–hole recombination rate was decreased, and the photocurrent was enhanced by approximately 5.5 times. Wang et al. used Ar plasma etching to reduce the surface oxide thickness, facilitating electron–hole separation and improving the photocurrent [3]. Liu et al. used three layers, namely a hole-storage layer, a blocking layer, and a cocatalyst to modulate the Ta_3_N_5_ surface, achieving a near-theoretical photocurrent density of 12.1 mA·cm^−2^ at 1.23 V vs. RHE [4]. Those results verify that suitable surface modification can enhance the electron–hole separation efficiency and injection efficiency of tantalum nitride, and even push them close to the theoretical ideal values of Ta_3_N_5_. In recent years, strategies for constructing surfaces with dipole moments to boost photogenerated charge-carrier separation have been widely reported. Joliat et al. introduced phosphonic acid (PA, H_3_PO_3_) into the photoelectrode, which enhances the dipole effect at the semiconductor interface and significantly improves both photovoltage and photocurrent density [5]. Yang et al. prepared a TiO_2_/BaTiO_3_ core/shell nanowire (NW) photoanode for photoelectrochemical water splitting. The BaTiO_3_ thin layer exhibits a ferroelectric polarization after the photoanode undergoes external electric field poling, which increases the band bending and promotes charge separation on the surface [6]. Polyelectrolytes including poly(diallyl-dimethylammonium chloride) (PDDA) and poly(sodium-p-styrenesulfonate) (PSS) possess intrinsic dipole moments. They can enhance charge separation at the photoelectrode surface and interface [7]. However, considering that these polyelectrolyte-modified layers are only on the nanometer scale, their working mechanism remains poorly understood.

In this work, we employ polyelectrolytes (PDDA and PSS) and Co(OH)_x_ for Ta_3_N_5_ pyramid array electrodes, representing an optimization strategy that addresses both surface modification and morphology control. The selected Ta_3_N_5_-PDDA_3_PSS_3_-Co(OH)_x_ pyramid array electrodes delivered the highest photocurrent densities of 7.7 mA cm^−2^ at 1.23 V vs. RHE and 11.9 mA cm^−2^ at 1.4 V vs. RHE. Further photoelectrochemical analysis shows that the polyelectrolyte layer functions only in the presence of a cocatalyst, indicating that polyelectrolytes facilitate hole transport while Co(OH)_x_ accelerates surface kinetics.

## 2. Results and Discussion

**Figure 1:**
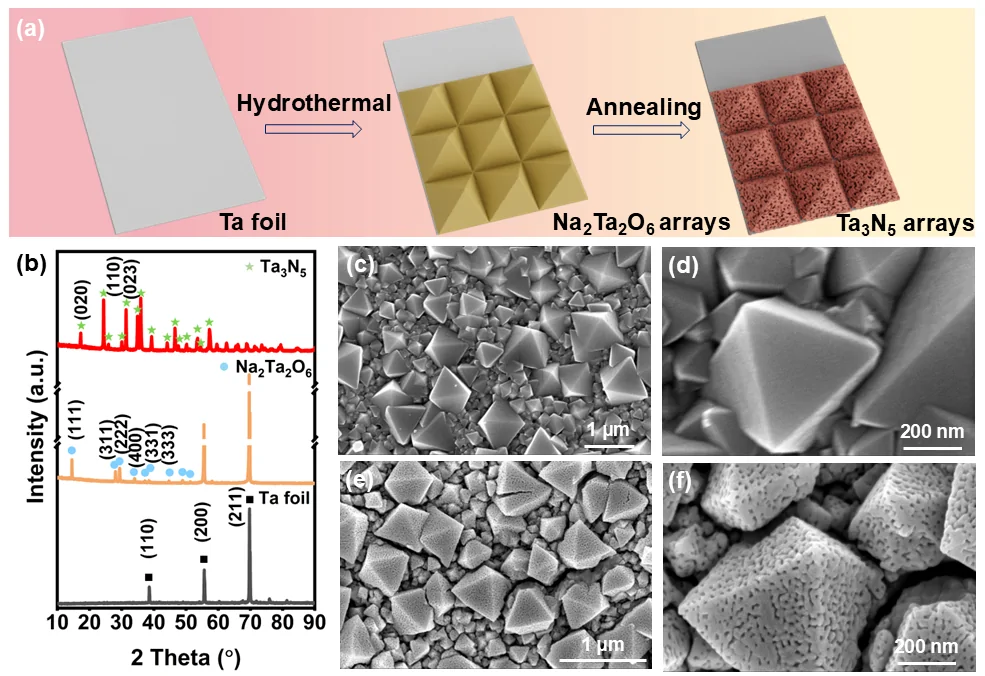
(**a**) Synthetic process for the Ta_3_N_5_ pyramid arrays, and (**b**) corresponding XRD patterns. SEM images of as-hydrothermally prepared (**c**,**d**) Na_2_Ta_2_O_6_ and (**e**,**f**) as-nitride Ta_3_N_5_ at low and high magnifications.

As shown in Figure 1a, we prepared pyramid arrays on Ta foil via the hydrothermal method. The foil was then annealed in air and in an ammonia atmosphere, respectively. We characterized the electrodes’ crystal structure using X-ray diffraction (XRD) (Figure 1b). The diffraction peaks of the electrode after hydrothermal treatment were located at 14.6°, 28.1°, 29.4°, 34.2°, 37.3° and 44.8° which are well matched with the (111), (311), (222), (400), (331) and (333) crystal planes of Na_2_Ta_2_O_6_ (JCPDF No. 97-000-2309, *Fd-3m* space group). Furthermore, the additional peaks at 38.5°, 55.6°, and 69.8° observed for the Na_2_Ta_2_O_6_ electrode belong to the crystal planes of Ta (JCPDF No.00-004-0788) originating from the substrate. To evaluate the preferential crystalline growth of this Na_2_Ta_2_O_6_ electrode, the texture coefficient (TC) was calculated using Equation (1). As shown in Figure S1a,b (Supporting Information), the (111), (311), and (331) planes exhibit TC values greater than 1, indicating preferred growth orientations, with the (111) plane showing the highest value of 1.67. To our knowledge, exposure of (111) or {111} [3] planes promotes the formation of an octahedral crystal (Figure S1c). They possess low surface energy during crystal growth and are thus retained in the final morphology, as predicted by the Gibbs-Wulff theorem. This theory was also applied to other crystals with the cubic space group, such as Fe_3_O_4_, Cu_2_O, and Ag_3_PO_4_ [8,9,10]. The morphology was further confirmed by SEM characterization. As illustrated in Figure 1c,d, the surface of the Na_2_Ta_2_O_6_ electrode, characterized by scanning electron microscopy (SEM), is covered with pyramid-like arrays composed of octahedral units, with edge lengths ranging from 0.2 to 0.9 μm. In the following nitridation process, the Ta_3_N_5_ pyramid arrays were derived from the Na_2_Ta_2_O_6_ template. The diffraction peaks of the electrode matched only with standard Ta_3_N_5_ (JCPDF No. 97-001-6253, *Cmcm* space group), indicating the successful synthesis of Ta_3_N_5_ crystals. The SEM image of the electrode still shows a pyramidal morphology, indicating that the shape and size of Na_2_Ta_2_O_6_ are largely maintained after nitridation (Figure 1e,f). In contrast, the Ta_3_N_5_ surface was full of pores, likely due to the change in crystal phase. These pores may help the electrode improve light-absorption efficiency and provide more active sites.

**Figure 2:**
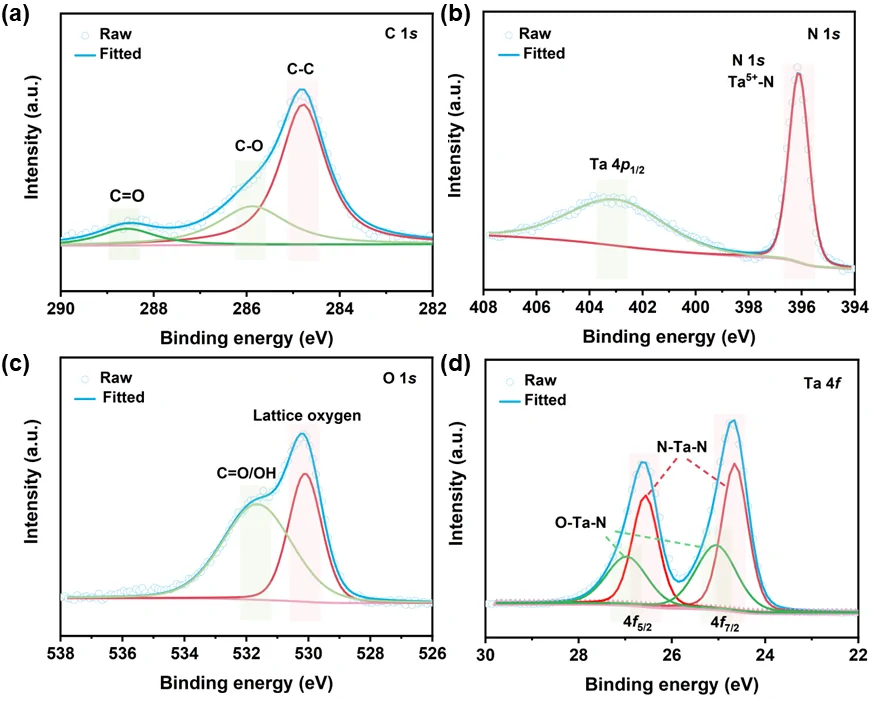
High-resolution XPS spectra of (**a**) C 1s, (**b**) N 1s, (**c**) O 1s, and (**d**) Ta 4f for original Ta_3_N_5_ pyramid arrays.

The surface state and composition of the original Ta_3_N_5_ pyramid arrays were confirmed by X-ray photoelectron spectroscopy (XPS). For the C 1s high-resolution spectrum (Figure 2a), the peak was deconvoluted into three peaks: 284.8 eV (C-C), 285.9 eV (C-O), and 288.6 eV (C=O). We used the C-C binding energy as the calibration reference. In the N 1s spectrum (Figure 2b), only one sharp peak appears at 396.1 eV, which is assigned to Ta^5+^-N species as reported in previous work [11]. In Figure 2c, the O 1s spectrum shows two peaks, with the binding energy at 531.7 eV assigned to C=O or a hydroxyl group (-OH), and the 530.1 peak related to lattice oxygen in Ta_3_N_5_ [12]. The Ta 4f spectrum exhibited two sets of pairwise peaks (Figure 2d); the core peaks are centered at 24.6 and 26.5 eV, corresponding to Ta 4f_7/2_ and Ta 4f_5/2_ of Ta-N, with a spin-orbit separation of 1.9 eV [13,14]. The other pair of peaks, located at 25.0 and 26.9 eV, are lower than the Ta-O binding energies reported for both Ta_2_O_5_ and NaTaO_3_ [15]. Therefore, these peaks cannot be attributed to Ta-O species, but rather to O-Ta-N moieties similar to those in TaON [16,17]. Combined with the XRD results confirming the formation of phase-pure Ta_3_N_5_, it can be inferred that the surface suffers from incomplete nitridation or partial oxidation under air exposure.

**Figure 3:**
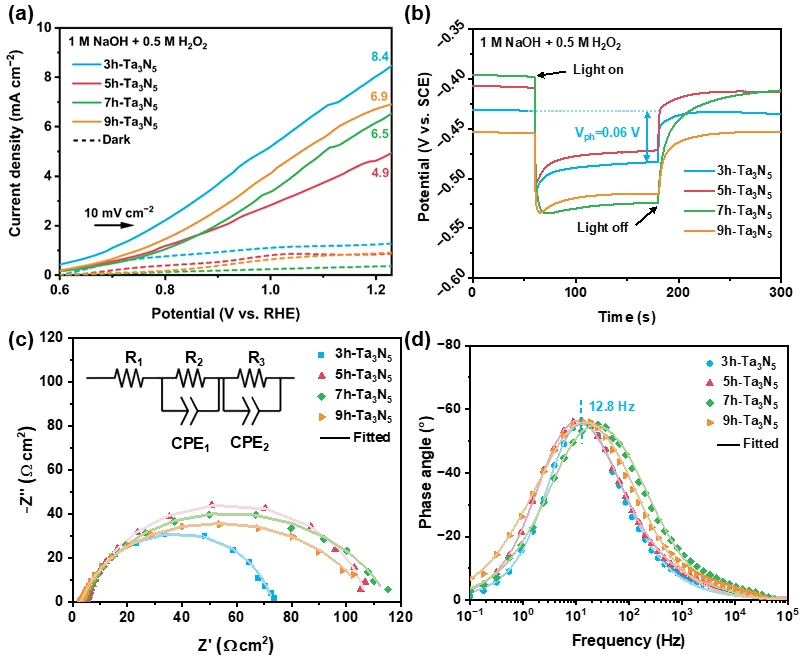
Photoelectrochemical characterization of 3h-Ta_3_N_5_, 5h-Ta_3_N_5_, 7h-Ta_3_N_5_, and 9h-Ta_3_N_5_. (**a**) J-V curves under dark (dashed lines) and illuminated (solid lines) conditions at a scan rate of 10 mV s^−1^. (**b**) OCP plots. (**c**) EIS Nyquist plots with the inserted equivalent circuit and (**d**) Bode curves at 1.23 V vs. RHE under illumination. Electrolyte: 1 M NaOH and 0.5 M H_2_O_2_ (pH = 13.6). Light: AM 1.5 G illumination (100 mW cm^−2^).

To ensure the electrodes were fully nitrided, the photoelectrochemical properties of samples with different nitriding durations in electrolytes containing H_2_O_2_ as a sacrificial agent under simulated solar irradiation. Figure 3a shows the photocurrent-potential (*J-V*) curves under dark and illuminated conditions, with results for shorter nitriding durations shown in Figure S2. Among all the photoanodes, the 3h-Ta_3_N_5_ electrode exhibits a maximum photocurrent density of 8.4 mA cm^−2^ at 1.23 V vs. RHE, which is 7.2 mA cm^−2^ after subtracting the dark current. Open-circuit potential (OCP) tests were performed under both illuminated (OCP_light_) and dark (OCP_dark_) conditions (Figure 3b). As expected, due to the small band bending in the sacrificial agent H_2_O_2_, 3h-Ta_3_N_5_ exhibits a similar photovoltage (V_ph_ = $\left|{\mathrm{OCP}}_{\mathrm{light}}-{\mathrm{OCP}}_{\mathrm{dark}}\right|$) to 5h-Ta_3_N_5_ and 7h-Ta_3_N_5_, at approximately 0.06 V. And it indicates that these samples possess a similar driving force in the electrolyte. Photoelectrochemical impedance spectroscopy (PEIS) plots further distinguished them, as shown in Figure 3c,d. The Nyquist plots were recorded at 1.23 V vs. RHE under illumination and fitted by the inserted “Voigt circuit”. Typically, R_1_ denotes the series resistance, R_2_ and CPE_2_ represent the resistance and constant phase element corresponding to the bulk (space charge region) at high frequency, while R_3_ and CPE_3_ represent elements associated with semiconductor–electrolyte charge transfer (Helmholtz layer) at low frequency [18]. The fitted data R_2_ for 3h-Ta_3_N_5_, 5h-Ta_3_N_5_, 7h-Ta_3_N_5_, and 9h-Ta_3_N_5_ are 15.0, 100.0, 92.5, and 90.7 Ω, respectively. It reveals that the advantage of 3h-Ta_3_N_5_ is its low bulk resistance and slightly higher resistance at the semiconductor–electrolyte interface (see Table S1 for more details). Notably, 3h-Ta_3_N_5_ also exhibits the minimum peak frequency of 12.8 Hz in the Bode plot (Figure 3d). Since the peak frequency is inversely related to the lifetime of photogenerated carriers, this finding further indicates that 3h-Ta_3_N_5_ has lower charge-migration resistance and a reduced electron–hole recombination rate [19].

To further enhance the PEC performance of 3h-Ta_3_N_5_ (denoted as Ta_3_N_5_), the polyelectrolytes (PSS (−) and PDDA (+)) were modified by the LBL method. This modified structure was evaluated by high-resolution transmission electron microscopy (HR-TEM) and energy-dispersive X-ray spectroscopy (EDX) mapping as follows. In Figure S3, the original Ta_3_N_5_ displays distinct lattice fringes of 0.355 nm, corresponding to the (110) plane of orthorhombic Ta_3_N_5_, which is consistent with the XRD result. After modification with three layers of PDDA and three layers of PSS (Ta_3_N_5_-PDDA_3_PSS_3_). The surface of Ta_3_N_5_ is covered by a layer of a thin amorphous film (Figure S3b). EDX mapping confirms the coexistence of Ta, N, and S, with S derived from the benzenesulfonate moiety of PSS (Figures S3c–f and S4a,b). PDDA and PSS are strong polyelectrolytes that fully dissociate over a wide pH range [19]. When Ta_3_N_5_ is immersed in their solution, they spontaneously adsorb onto the Ta_3_N_5_ surface via electrostatic interactions, as we demonstrated in our previous work [7]. These results indicate that the polyelectrolytes were successfully modified onto Ta_3_N_5_. Moreover, we used ultraviolet-visible diffuse-reflectance spectroscopy (UV-Vis DRS) to examine the electrode’s light-absorption characteristics (Figure S5a,b). The Ta_3_N_5_ electrode, before and after modification with PDDA_3_/PSS_3_, exhibits similar absorption band edges at around 616 nm. Both the bandgap (*E*_g_) of Ta_3_N_5_ and Ta_3_N_5_-PDDA_3_/PSS_3_ are 2.05 eV, based on the previous study and the standard solar spectrum at AM 1.5 G (NREL, units: mW cm^−2^ nm^−1^), [20,21] giving a theoretical maximum photocurrent density of 13.5 mA cm^−2^.

**Figure 4:**
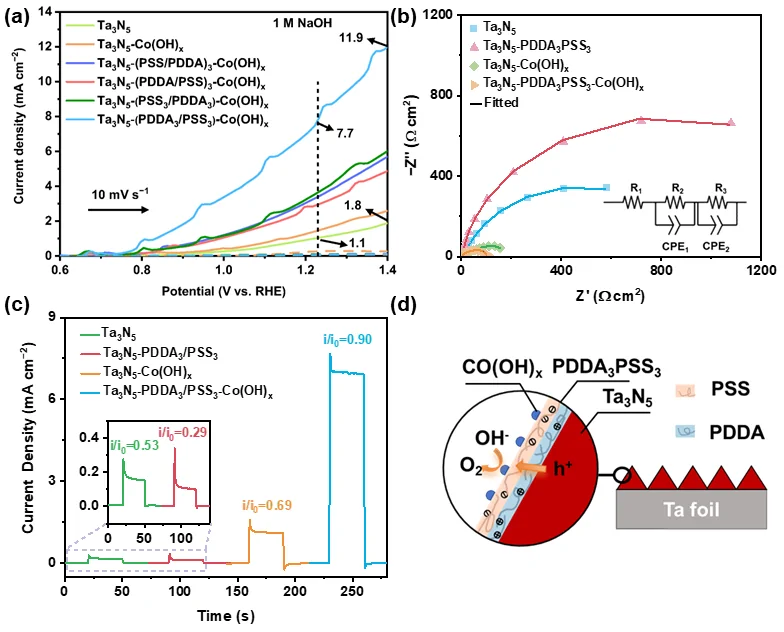
Photoelectrochemical characterization of Ta_3_N_5_-based photoelectrodes. (**a**) J-V curves under dark and light conditions at a scan rate of 10 mV s^−1^, (**b**) original and fitted EIS Nyquist plots with the inserted equivalent circuit, and (**c**) analyzed transient photoresponse. (**d**) A scheme to describe the behavior of carrier migration in Ta_3_N_5_ after being modified with a polyelectrolyte layer and catalysis. Electrolyte: 1 M NaOH (pH = 13.6). Light: AM 1.5G illumination (100 mW cm^−2^).

To investigate the polyelectrolyte-modified electrodes, a series of PEC measurements was conducted. As illustrated in Figure S6a, linear sweep voltammetry (LSV) curves of Ta_3_N_5_ and Ta_3_N_5_ with different polyelectrolyte modification sequences were measured in 1 M NaOH under simulated one-sun illumination. PDDA and PSS are positively and negatively charged, respectively, as shown in the insert. The results show that the photocurrent density of pristine Ta_3_N_5_ is approximately 2 mA cm^−2^ at 1.4 V vs. RHE. The photocurrent of the four polyelectrolyte-modified electrodes shows only a slight enhancement compared with the bare sample. Furthermore, Ta_3_N_5_ with different polyelectrolyte modifications has no advantage in either the chronoamperometric photocurrent or EIS measurements (Figure S6b,c). After loading a catalyst onto the corresponding electrodes, the photocurrent increased significantly. As shown in Figure 4a, Ta_3_N_5_-PDDA_3_PSS_3_-Co(OH)_x_ exhibits a photocurrent density of 7.7 mA cm^−2^ at 1.23 V vs. RHE and 11.9 mA cm^−2^ at 1.4 V vs. RHE, representing a competitive performance compared with previous studies (Table S2). The photocurrent is enhanced by over 7-fold compared with Ta_3_N_5_. Figure S7a shows the corresponding chronoamperometric photocurrent measured at 1.23 V vs. RHE. The optimized electrode Ta_3_N_5_-PDDA_3_PSS_3_-Co(OH)_x_ shows a similar current density at the same potential as that in the LSV curve, while the pristine Ta_3_N_5_ electrode shows a significantly reduced current. Overall, the results show that the optimized electrode is more stable than the pristine one. On the other hand, it shows that the surface modification acts as a hole-transport layer. A higher scan rate in LSV measurements leads to increased photocurrent density. The total current *I*_total,LSV_ at the semiconductor–electrolyte interface has two components: the faradaic current *I*_faradaic_, arising from the water oxidation reaction, and the double-layer capacitive current *I*_double layer_ associated with interface charge separation or non-faradaic capacitive current [22,23]. In contrast, the transient photocurrent *I*_total,trans_ is recorded under steady-state conditions and thus reflects only *I*_faradaic_. The similarity between *I*_total,LSV_ and *I*_total,trans_ suggests highly efficient charge transport, which can be attributed to the PDDA_3_PSS_3_ interlayer acting as a hole transport layer [24]. The EIS measurements were subsequently used to evaluate charge separation performance. The diameter of the Nyquist plot for Ta_3_N_5_-PDDA_3_PSS_3_-Co(OH)_x_ is much smaller than that of other samples, including the bare Ta_3_N_5_ (Figure S7b).

To reveal the synergistic effect between the polyelectrolyte PDDA_3_PSS_3_ layer and the cocatalyst Co(OH)_x_, the fitted data of Ta_3_N_5_, Ta_3_N_5_-PDDA_3_PSS_3_, Ta_3_N_5_-Co(OH)_x_, and Ta_3_N_5_-PDDA_3_PSS_3_-Co(OH)_x_ are summarized in Table S3 and presented in Figure 4b. The corresponding equivalent circuit is shown in the inset of Figure 4, where R_2_ and R_3_ represent the resistance for charge transfer in the bulk and charge injection. After modification with Co(OH)_x_, the radii of the semicircles for Ta_3_N_5_-Co(OH)_x_ and Ta_3_N_5_-PDDA_3_PSS_3_-Co(OH)_x_ decrease significantly. Furthermore, the R_2_ value of Ta_3_N_5_-PDDA_3_PSS_3_-Co(OH)_x_ is much smaller than that of Ta_3_N_5_-Co(OH)_x_, representing that the holes are much more readily transported to the electrode surface upon polyelectrolyte modification. It can therefore be inferred that the polyelectrolyte and cocatalyst work synergistically to enhance the photocurrent. Analysis of the transient photocurrent responses revealed that Ta_3_N_5_-PDDA_3_PSS_3_-Co(OH)_x_ exhibits an i/i_0_ ratio of 0.9, the only value close to 1 (Figure 4c). Here, i and i_0_ represent steady-state and initial peak photocurrent densities, respectively. This demonstrates efficient transport of charge carriers to the surface during the water oxidation reaction. Thus, the PEC analyses show that Ta_3_N_5_-PDDA_3_PSS_3_-Co(OH)_x_ promotes interfacial kinetics due to the synergistic effects of polyelectrolyte modification and Co(OH)_x_ catalysis, as shown in Figure 4d.

## 3. Materials and Methods

### 3.1. Chemicals and Reagents

The Ta sheet (≥99.95%, 0.3 mm) was purchased from ZhongNuo Advanced Material Technology Co., Ltd. (Beijing, China). Sodium hydroxide (NAOH, ≥98%), and poly(diallyldimethylammonium chloride) (PDDA, M_w_ < 100,000, 38 wt. % in water, 100–200 cP (25 °C)) were purchased from Shanghai Aladdin Bio-Chem Technology Co., Ltd. (Shanghai, China). Poly(sodium-p-styrenesulfonate) (PSS, M_w_ ~ 70000) was purchased from Shanghai Macklin Biochemical Co., Ltd. (Shanghai, China). Acetone, ethanol, and hydrogen peroxide (30%) were purchased from Xilong Chemical Factory (Shantou, China). Deionized water (DI water, >18 MΩ cm resistivity) was produced by a Water Purification System of Heal Force (Shanghai, China).

### 3.2. Synthesis of Ta_3_N_5_ Pyramid Arrays Photoelectrode

The Ta_3_N_5_ pyramid arrays were synthesized by a three-step process. Firstly, the Ta sheet was prepared by the hydrothermal method. Briefly, the Ta sheet was ultrasonically cleaned in acetone, ethanol, and water for 20 min each in sequence. The Ta sheet was transferred to a 100 mL Teflon vessel containing 0.05 M NaOH and maintained at 200 °C for 12 h (Na_2_Ta_2_O_6_ pyramid arrays). Secondly, the samples were annealed in a muffle furnace at 500 °C for 3 h with a heating and cooling rate of 2 °C min^−1^. Thirdly, the film was further calcined in ammonia (NH_3_) at 950 °C for 3 h to obtain Ta_3_N_5_ pyramid arrays. For further evaluation, the electrodes annealed in ammonia for 3, 5, 7, and 9 h were designated as 3h-Ta_3_N_5_, 5h-Ta_3_N_5_, 7h-Ta_3_N_5_, and 9h-Ta_3_N_5_, respectively.

### 3.3. Layer-by-Layer Modification of PDDA/PSS on Ta_3_N_5_ Photoelectrode

Polyelectrolytes PDDA and PSS were deposited on the Ta_3_N_5_ photoelectrode by the layer-by-layer (LBL) method. Before deposition, the Ta_3_N_5_ pyramid array photoelectrode was immersed in a solution of HF, HNO_3_, and H_2_O (1:2:7) for 30 s. It was then thoroughly rinsed with deionized water and dried with an air blower. Afterward, the sample was immersed in a 1.5% (*v*/*v*) PDDA solution or a 5 mg mL^−1^ PSS solution for 5 min, then rinsed with DI water for 150 s. This step represented one deposition cycle, corresponding to a single polyelectrolyte layer, and the cycle was repeated based on the electrode designation.

### 3.4. Modification of Co(OH)_x_ Cocatalysts

The modification of Co(OH)_x_ cocatalysts was carried out using the chemical bath deposition (CBD) method [25]. The prepared photoelectrode was immersed in a mixed solution of 0.1 M CoSO_4_ and 0.1 M NaOH, and then maintained at 40 °C for 20 min. After that, the sample was rinsed with DI water and dried.

### 3.5. Material Characterization

The crystal structure and phase information of the electrodes were analyzed using an X-ray diffractometer (XRD, Bruker, Karlsruhe, Germany, D8 Advance) with monochromatic Cu Kα radiation. To evaluate the preferential crystalline growth of this Na_2_Ta_2_O_6_ electrode, the texture coefficient (TC) was calculated using the following Equation (1) [26]:

(1) $$\mathrm{TC}(hkl)\;=\;\frac{\frac{I(hkl)}{I_{0}(hkl)}}{\frac{1}{N}\sum_{N}\frac{I(hkl)}{I_{0}(hkl)}}$$

where TC(hkl) is the TC of the specific crystallographic plane (hkl), I(hkl) is the measured relative peak intensity, $I_{0}(hkl)$ is the standard relative peak intensity taken from the JCPDF card of Na_2_Ta_2_O_6_, and N is the number of reflections considered. The morphologies of the electrodes were recorded by scanning electron microscopy (SEM, Verios G4 UC, Thermo Scientific, Hillsboro, OR, USA) and high-resolution transmission electron microscopy (HR-TEM, Thermo Scientific, Talos F200X G2). The surface chemical composition of the sample was measured using X-ray photoelectron spectroscopy (XPS, Kratos, Axis Supra). Peak deconvolution was performed using XPSpeak software (Version 4.1), with all peaks fitted at a G/L ratio of 80 and calibrated to the C-C peak (284.8 eV). The optical properties of the sample were measured using an ultraviolet–visible diffuse reflectance spectroscopy (UV–Vis DRS, Beijing Persee, Beijing, China, TU-1901).

### 3.6. Photoelectrochemical (PEC) Measurements

All PEC tests were recorded in a three-electrode system using a potentiostat (Zennium Zahner, Kronach, Germany) under simulated AM 1.5G solar light irradiation (100 mW cm^−2^, Aulight, Beijing, China). The sample was used as the working electrode (active area: 1 cm^2^), with an Hg/HgCl_2_ electrode as the reference electrode, and a platinum plate as the counter electrode. The electrolyte was 1 M NaOH (pH = 13.6) and the potential was corrected to the RHE scale according to the Nernst Equation (2):

(2) $$E_{RHE}=E_{{Hg/HgCl}_{2}}^{\theta }+E_{{Hg/HgCl}_{2}}+0.059\times pH$$

where $E_{{Hg/HgCl}_{2}}^{\theta }$ is about 0.241 and $E_{{Hg/HgCl}_{2}}$ represent the applied bias potential in the PEC cell. Photoelectrochemical impedance spectroscopy (PEIS) was recorded at 1.23 V vs. RHE under illumination, with a frequency range from 0.1 Hz to 100 kHz.

## 4. Conclusions

In summary, we established a highly regulated Ta_3_N_5_-PDDA_3_PSS_3_-Co(OH)_x_ pyramid array photoelectrode for PEC water splitting. We fabricated the regular Ta_3_N_5_ pyramid arrays using cubic Na_2_Ta_2_O_6_ as a self-template. The preferential (111) orientation of Na_2_Ta_2_O_6_ contributes to the formation of this uniform pyramidal structure. The best Ta_3_N_5_ photoanode exhibits a photocurrent density of 7.2 mA cm^−2^ at 1.23 V vs. RHE, in an electrolyte containing the sacrificial agent H_2_O_2_. Moreover, after surface modification with polyelectrolytes and catalysis, the Ta_3_N_5_-PDDA_3_PSS_3_-Co(OH)_x_ electrode delivers 7.7 mA cm^−2^ at 1.23 V vs. RHE and 11.9 mA cm^−2^ at 1.4 V vs. RHE in 1 M NaOH under simulated solar illumination. More PEC measurements reveal that the polyelectrolyte layer facilitates hole transport, while Co(OH)_x_ accelerates surface reaction kinetics. This study provides a feasible route for morphology and surface modulation, and we will further investigate stability improvements.

## Data Availability

The data used to support the findings of this study are available from the corresponding author upon request.

## Supplementary Materials

The following supporting information can be downloaded at: https://www.mdpi.com/article/10.3390/ijms27188281/s1. References [27,28,29,30,31,32,33,34,35,36] are cited in supplementary materials.
