# Supplementary material for: Layer-by-Layer Assembled Polyelectrolytes on Ta3N5 Pyramid Arrays for Efficient Photoelectrochemical Water Splitting
Source: Int J Mol Sci. 2026 Sep 17;27(18):8281. doi: 10.3390/ijms27188281 (PMC13607712; doi:10.3390/ijms27188281)
Supplement: Supplementary file 1 [file ijms-27-08281-s001.zip › ijms-4539533-supplementary.pdf]

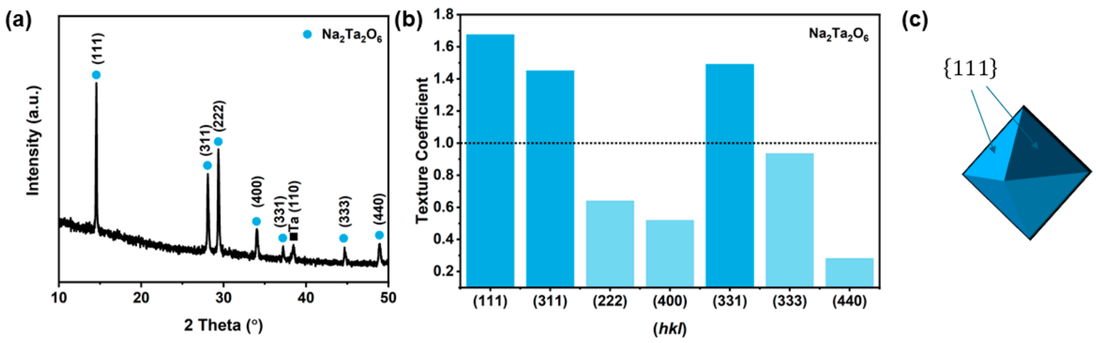

Figure S1 (a) The XRD pattern of $NaTaO_3$ in the range of 10° to 50°. (b) The texture coefficient of selected dominant diffraction peaks of $NaTO_3$ photoelectrode. (c) Schematic models of the crystal structure with exposed [1] face planes.

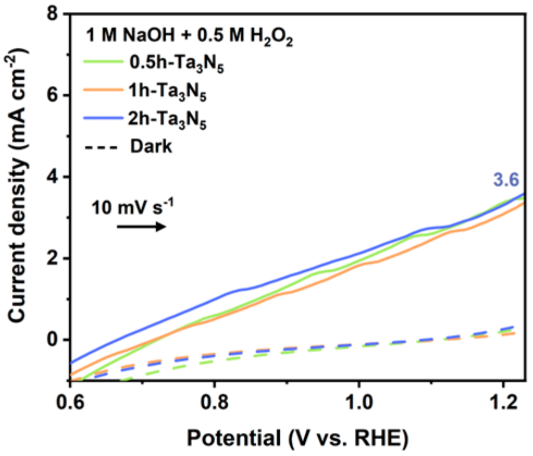

Figure S2 *J*-*V* curves of Ta_3N_5 with different nitriding durations for 0.5 - 2 h (0.5h-Ta_3N_5, 1h-Ta_3N_5, and 2h-Ta_3N_5) under dark (dashed lines) and illuminated (solid lines) conditions. Electrolyte: 1 M NaOH and 0.5 M H_2O_2 (pH = 13.6). Light: AM 1.5G illumination.

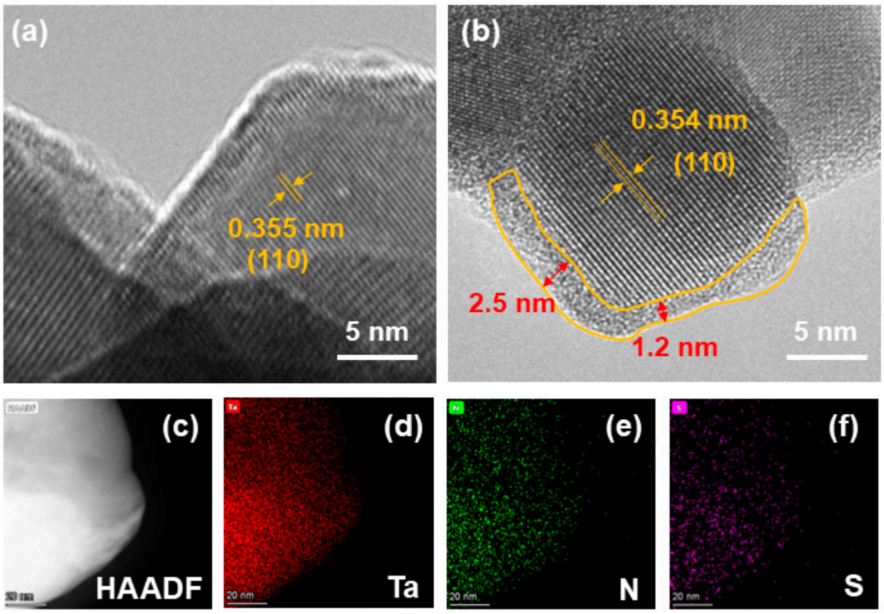

Figure S3 TEM image of (a) Ta_3N_5 and (b) Ta_3N_5-$PDDA_3PSS_3$. (c-d) EDX mapping imagines of Ta_3N_5-$PDDA_3PSS_3$.

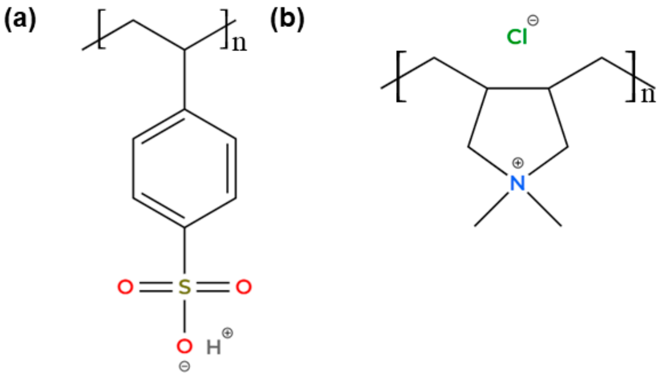

Figure S4 Chemical structure of polyelectrolytes PSS and PDDA.

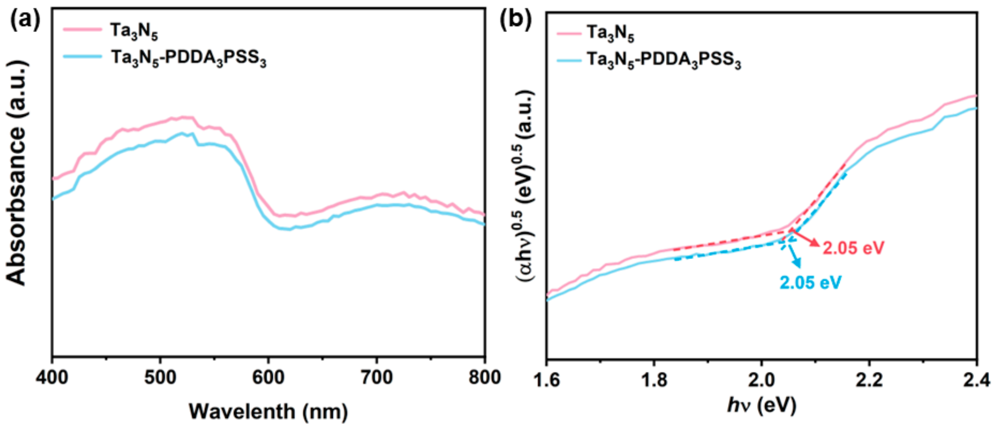

Figure S5 (a) UV-Vis DRS spectra and (b) indirect bandgap tauc plots of Ta_3N_5 and Ta_3N_5-PDDA$_3$PSS$_3$ electrodes.

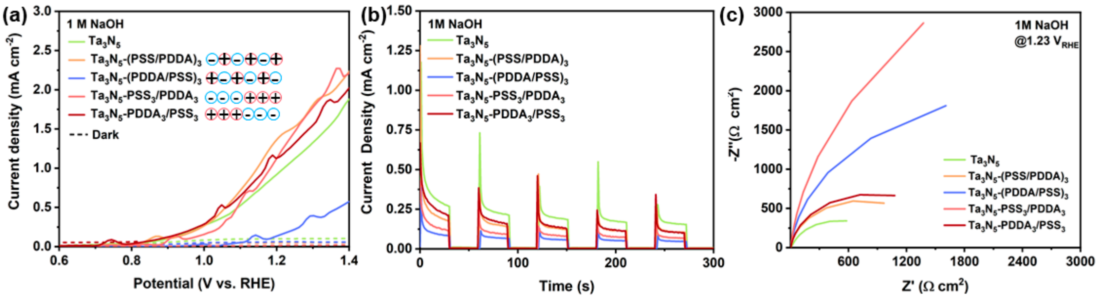

Figure S6 Photoelectrochemical characterization of Ta_3N_5-based photoelectrodes. (a) *J-V* curves under dark and light conditions, (b) transient photoresponse at 1.23 V vs. RHE, (c) EIS Nyquist plots at 1.23 V vs. RHE under illumination. Electrolyte: 1M NaOH (pH=13.6). Light: AM 1.5G illumination.

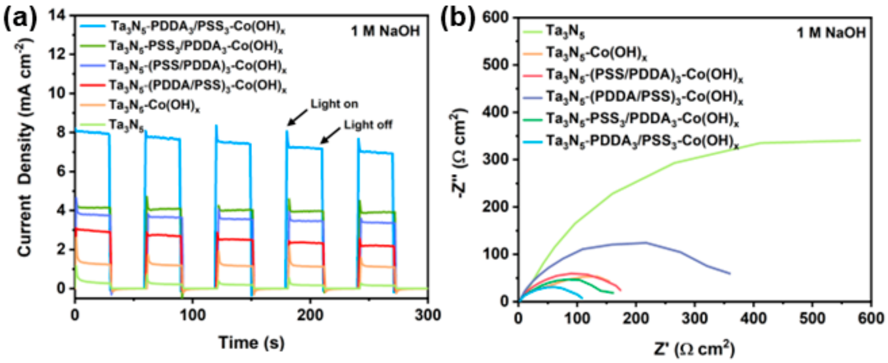

Figure S7 Photoelectrochemical characterization of Ta_3N_5-based photoelectrodes. (a) chronoamperometric photocurrent at 1.23 V vs. RHE and (b) EIS Nyquist plots at 1.23 V vs. RHE under illumination. Electrolyte: 1 M NaOH (pH=13.6). Light: AM 1.5G illumination.

Table S1 EIS fitting results of 3h-Ta_3N_5, 5h-Ta_3N_5, 7h-Ta_3N_5, 9h-Ta_3N_5.

Photoelectrode	R_1 (Ω)	R_2 (Ω)	R_3 (Ω)
3h-Ta_3N_5	4.1	15.0	54.7
5h-Ta_3N_5	4.1	100	3.3
7h-Ta_3N_5	3.6	92.5	19.6
9h-Ta_3N_5	2.9	90.7	14.3

Table S2 Comparison of recently published studies concerning Ta_3N_5-based photoelectrodes for water splitting.

Material structure[a-c]	Surface modification	Co-catalysis	electrolyte	Photocurrent density (mA cm^{-2} at 1.23 V_{RHE})[d]
Ta_3N_5/Si film[2]	-	-	0.1 M NaPi buffer + 0.1 M $K_4[Fe(CN)_6]$	3.86
Ta_3N_5 film[3]	-	-	1 M KOH + 0.1 M $K_4[Fe(CN)_6]$	4.0
P:Ta_3N_5 film[4]	-	-	1 M KOH + 0.2 M H_2O_2	4.3
Ta_3N_5/GaN/ Al_2O_3[5]		$NiFeO_x$	1 M KOH	7.4
Ta_3N_5 NAs[6]	-	$Co(OH)_x$/ CoOOH	1 M NaOH	2.96
Ta_3N_5/ Ta_5N_6 film[7]	-	NiCoFe-B_i	1 M KOH	9.9
Ta_3N_5 NT/NP[8]	-	$Co(OH)_x$	1 M KOH	8.73
Ta_3N_5 NT[9]	-	NiFe	1 M KOH	11.2

Ta_3N_5/NbN_x/n-Si film[10]	-	CoO_x	1 M KOH	6.64
Ta_3N_5/TiN film[11]	CPF-TTB	$NiFeO_x$	1 M KOH	9.12
Ta_3N_5 pyramid arrays (this work)	-	-	1 M NaOH + 0.5 M H_2O_2	7.2
Ta_3N_5 pyramid arrays (this work)	$PDDA_3PSS_3$	$Co(OH)_x$	1 M NaOH	7.7

a: NT represents nanotube. b: NP represents nanoparticle. c: NAs represents nanoarchitectures. d: light source for all samples is AM 1.5G.

Table S3 EIS fitting results of Ta_3N_5, Ta_3N_5-$PDDA_3PSS_3$, Ta_3N_5-$Co(OH)_x$, Ta_3N_5-$PDDA_3PSS_3$-$Co(OH)_x$.

Photoelectrode	R_1 (Ω)	R_2 (Ω)	R_3 (Ω)
Ta_3N_5	2.3	935.5	7.6
Ta_3N_5-$PDDA_3PSS_3$	3.3	712.1	861.5
Ta_3N_5-$Co(OH)_x$	2.1	136.4	53.6
Ta_3N_5-$PDDA_3PSS_3$-$Co(OH)_x$	2.4	45.4	56.3

Reference

(1) Abbas, M. A.; Kim, T.-Y.; Lee, S. U.; Kang, Y. S.; Bang, J. H. Exploring Interfacial Events in Gold-Nanocluster-Sensitized Solar Cells: Insights into the Effects of the Cluster Size and Electrolyte on Solar Cell Performance. *Journal of the American Chemical Society* **2016**, *138* (1), 390–401. DOI: 10.1021/jacs.5b11174.
(2) Chang, C.; Lai, B.; Jiang, C. Efficient Ta_3N_5 Photoanodes via Interface Engineering of Bixbyite-Type Ta_2N_3 Precursors. *Small* **2025**, *21* (37), e05487. DOI: https://doi.org/10.1002/smll.202505487.
(3) Francisco, F. M. M.; Dias, P.; Mendes, A. Unlocking the potential of semi-transparent Ta_3N_5 photoelectrodes for high-performance, reproducible solar redox flow cells. *Journal of Materials Chemistry A* **2025**, *13* (20), 14601–14611. DOI: 10.1039/D4TA08136F.
(4) Dong, C.; Zhang, Y.; Bi, Y.; Li, Z.; Ding, Y. Band structure engineering of phosphorus doped Ta_3N_5 for efficient photoelectrochemical water oxidation. *Chinese Chemical Letters* **2025**, *36* (12), 111449. DOI: https://doi.org/10.1016/j.cclet.2025.111449.
(5) Higashi, T.; Nishiyama, H.; Nandal, V.; Pihosh, Y.; Kawase, Y.; Shoji, R.; Nakabayashi, M.; Sasaki, Y.; Shibata, N.; Matsuzaki, H.; et al. Design of semitransparent tantalum nitride photoanode for efficient and durable solar water splitting. *Energy & Environmental Science* **2022**, *15* (11), 4761–4775. DOI: 10.1039/D2EE02090D.
(6) Wang, Q.; Zhang, L.; Li, B.; Zhu, H.; Shi, J. 3D interconnected nanoporous Ta_3N_5 films for photoelectrochemical water splitting: thickness-controlled synthesis and insights into stability. *Science China Materials* **2021**, *64* (8), 1876–1888. DOI: 10.1007/s40843-020-1584-6.
(7) Liu, Y.; Fan, Z.; Li, R.; Mavrič, A.; Arčon, I.; Valant, M.; Kapun, G.; Zhang, B.; Feng, C.; Zhang, Z.; et al. Lattice-Matched Ta_3N_5/Nb_5N_6 Interface Enables a Bulk Charge Separation Efficiency of Close to 100%. *ACS Energy Letters* **2025**, *10* (4), 1911–1920. DOI: 10.1021/acsenergylett.5c00603.
(8) Zhang, X.; Guo, H. L.; Dong, G. J.; Zhang, Y. J.; Lu, G. X.; Bi, Y. P. Homostructural Ta_3N_5 nanotube/nanoparticle photoanodes for highly efficient solar-driven water splitting. *Applied Catalysis B-Environment and Energy* **2020**, *277*, 119217. DOI: 10.1016/j.apcatb.2020.119217.
(9) Dong, C.; Zhang, X.; Ding, Y.; Zhang, Y.; Bi, Y. Unveiling the high activity origin of NiFe catalysts decorated Ta_3N_5 photoanodes for oxygen evolution reaction. *Applied Catalysis B: Environmental* **2023**, *338*, 123055. DOI: https://doi.org/10.1016/j.apcatb.2023.123055.
(10) Kwon, H. R.; Yang, J. W.; Choi, S.; Cheon, W. S.; Im, I. H.; Kim, Y.; Park, J.; Lee, G. H.; Jang, H. W. Low Onset-Potential Z-Scheme Ta_3N_5-Based Photoanode with Enhanced Light Harvesting and Charge Transport. *Advanced Energy Materials* **2023**, *14* (5), 2303342. DOI: 10.1002/aenm.202303342.
(11) Yang, J. W.; Kwon, H. R.; Ji, S. G.; Kim, J.; Lee, S. A.; Lee, T. H.; Choi, S.; Cheon, W. S.; Kim, Y.; Park, J.; et al. Conjugated Polythiophene Frameworks as a Hole-Selective Layer on Ta_3N_5 Photoanode for High-Performance Solar Water Oxidation. *Advanced Functional Materials* **2024**, *34* (34), 2400806. DOI: 10.1002/adfm.202400806.
